# [Retracted] Metastasis‑associated protein 2 promotes the metastasis of non‑small cell lung carcinoma by regulating the ERK/AKT and VEGF signaling pathways

**DOI:** 10.3892/mmr.2025.13454

**Published:** 2025-02-06

**Authors:** Bin Zhang, Feng Tao, Hao Zhang

Mol Med Rep 17: 4899–4908, 2018; DOI: 10.3892/mmr.2018.8535

Following the publication of this paper, it was drawn to the Editor's attention by a concerned reader that the migration assay data shown in Fig. 2D on p. 4904 were strikingly similar to data that had already been published in another article in the journal *Oncotarget* written by different authors at different research institutes.

Owing to the fact that the contentious data in the above article had already been published prior to its submission to *Molecular Medicine Reports*, the Editor has decided that this paper should be retracted from the Journal. The authors were asked for an explanation to account for these concerns, but the Editorial Office did not receive a reply. The Editor apologizes to the readership for any inconvenience caused.

